# Editorial: Eating Behavior and Food Decision Making in Children and Adolescents

**DOI:** 10.3389/fpsyg.2021.818078

**Published:** 2022-01-17

**Authors:** Oh-Ryeong Ha, Seung-Lark Lim, Amanda S. Bruce, Travis D. Masterson, Shan Luo

**Affiliations:** ^1^Department of Psychology, University of Missouri – Kansas City, Kansas City, MO, United States; ^2^Department of Pediatrics, University of Kansas Medical Center, Kansas City, KS, United States; ^3^Center for Children's Healthy Lifestyles & Nutrition, Kansas City, MO, United States; ^4^Department of Nutritional Sciences, The Pennsylvania State University, University Park, PA, United States; ^5^Keck School of Medicine, University of Southern California, Los Angeles, CA, United States

**Keywords:** eating behavior, food choice, food preferences, dietary self-control, pediatric obesity, energy intake, children, adolescents

Obesity is a persistent societal health problem that has lasted for several decades. Developing healthy eating habits early in life is one of the major keys in establishing healthy lifestyles and preventing and treating obesity. Despite our current efforts to reduce the rate of obesity in children, trends project that more children and adolescents will be affected by obesity than previously seen. Encouragingly, scientists and health professionals have identified obesogenic characteristics of adults with obesity and have targeted those characteristics for obesity interventions. However, precursors or obesogenic characteristics of childhood obesity have not been fully identified. Early prevention and intervention could be crucial to reduce the prevalence of obesity and to improve the physical and mental health of individuals early in life. Therefore, more proactive approaches are needed. These could include promoting the development of healthy eating habits and identifying children at high risk of developing obesity to allow for early intervention prior to excessive weight gain. Further investigation on behavioral characteristics and neural mechanisms of pediatric obesity is warranted for effective obesity prevention and treatment.

Food decision-making is a complicated process involving an interplay between internal factors (e.g., interoceptive signals of hunger, dietary self-control) and external factors (e.g., family eating practices, food marketing). Healthy food choices are more demanding in children because food taste is a primary determinant, while food healthiness is far less considered. Dietary self-control does not work effectively at this developmental period to delay gratification resulting in food choices that satisfy immediate urges to eat energy-dense, highly palatable foods. In addition, appetitive traits reflect comparatively passive food experiences through parental eating behavior and feeding practices from prenatal periods. These challenges raise questions regarding how children learn to integrate all those signals, and how they learn to make healthy eating decisions and eventually build healthy eating habits. Therefore, this Research Topic aims to display the multifaceted mechanisms underlying the development of eating behavior and food choices from infancy to adolescence. The goal of this Research Topic is to illuminate effective strategies for promoting healthy eating and decreasing obesity in young populations.

## Overview of Contributions

### Food Choices

The first line of contributions focuses on the development of food choices in children and adolescents. Particularly, Eagleton et al. examined how the relative reinforcing value of high energy-dense foods (i.e., cookie) and that of low energy-dense foods (i.e., fruit) were related to obesity in children aged 3–5 years from low-income families attending Head Start. Their results suggested that developing the high reinforcing value of high energy-dense foods may contribute to obesity in boys with increasing age. Fuchs et al. conducted a within-subjects laboratory food intake study to examine how food decision-making processes influence energy intake and weight status in children aged 7–11 years. Their results suggested that children with a perseverance tendency in decision-making (i.e., repeating the same choice after a positive/rewarding experience) tend to consume high energy, which may contribute to weight gain. Serrano-Gonzalez et al. examined developmental changes of food perception (health and taste attributes) and food preferences in individuals aged 8–23 years. Their results suggested that children and adolescents with higher central adiposity are more likely to develop preferences for high-calorie foods and higher taste importance in food choices over time. Papantoni et al. conducted a longitudinal study of examining taste sensitivity, taste liking, dietary intake, and BMI percentiles of adolescents aged 14–16 years over 4 years. Their results suggested that adolescents with lower sweet taste sensitivity have a higher hedonic response to high-sugar foods, and those with high daily fat consumption are more likely to develop a preference for high-fat/high-sugar foods. In addition, food rejection was examined from a food decision-making perspective. Pickard et al. examined how food rejection would be associated with the development of taxonomic (e.g., bread and pasta) and thematic (e.g., bread and butter) food knowledge in children aged 3–6 years. Their results suggested that children are more likely to reject food with poor thematic food knowledge possibly due to a lack of exposure to various foods and associations. Foinant et al. examined how positive and negative reasoning of food health-related properties influences food choices in children aged 3–6 years. Results suggested that food neophobic children may make lower-risk food choices by generalizing negative reasoning to prevent potentially harmful consequences from consuming foods. These contributions suggest that children and adolescents who have developed taste- or reward-oriented decision-making would display high unhealthy food intake and/or weight gain. In addition, children who have developed negative reasoning about food or less exposure to different food would show high food rejection.

### Self-Regulation

The second line of contributions focuses on self-regulation and eating in the absence of hunger in children. Giuliani and Kelly investigated how dietary self-regulation (i.e., delay of gratification) and general self-regulation (i.e., attentional control and inhibitory control) would predict eating behaviors after 1 year in children aged 3–6 years. They reported that longer delay of gratification was related to high caloric intake in the absence of hunger, but the effect of delay of gratification was not significant when general self-regulation was controlled. Children with a poor delay of gratification and inhibitory control consumed the most calories. Philippe et al. examined how children's weight status, inhibitory control, and maternal feeding practices were associated with eating in the absence of hunger in children aged 2–6 years. They reported that children with higher BMI z-scores, lower inhibitory control, and higher maternal control were more likely to eat in the absence of hunger. Mason et al. proposed that decline in physical activity in middle childhood and poor inhibitory control would contribute to loss of control eating when children transit into adolescence. These contributions suggest that children who have developed poor dietary and/or general self-regulation are inclined to consume high energy in the absence of hunger, which could contribute to weight gain.

### Parents and Peers

The third line of contributions focuses on parental and peer influences on children's eating behaviors. Kong et al. reported that infants aged 9–15 months were exposed early to hyper-palatable foods (e.g., foods with high fat and sodium, high fat and sugar, or high carbohydrates and sodium) as they transitioned to adult foods offered by caregivers. Trevino et al. reported that emotional eating was more likely to be transmitted from parent to child with more use of maternal restrictive feeding practices and paternal emotion regulation, instrumental, and restrictive feeding practices in children aged 5–13 years. Solano-Pinto et al. reported that body dissatisfaction of boys aged 9–11 years was related to both own and maternal desire for ideal body image, approach to change through diet, and BMI, whereas body dissatisfaction of girls was only related to own factors, which may suggest that pressure to ideal body image would be internalized earlier in girls. Ziegler et al. reported on food-related behaviors in which adolescents aged 13–17 experience a range of autonomy. They also showed that factors such as time with peers and parental control can enhance or infringe on this autonomy. These contributions suggest that parental feeding practices, eating behaviors, and peer influence impact the development of eating habits in children and adolescents.

### Intervention

The last line of contributions in this issue focuses on intervention to promote healthy food choices. Porter et al. conducted food-specific inhibition training using a Go/No-Go task for children aged 4–10 years. Their results suggested that improving inhibitory control to energy-dense foods via food-specific inhibition training would promote healthy food choices. Ha et al. conducted a food advertising literacy intervention using cognitive and affective narratives presented after food commercials in videos in children aged 8–12 years. Their results suggested that enhancing resilience to food commercials by improving cognitive skepticism and critical thinking toward food advertising would promote less taste-oriented, more self-regulated eating decisions. These contributions suggest that providing intervention targeting to improve inhibitory control and cognitive defenses to external food cues would promote healthy food decision-making in children.

## Future Directions

Contributions to this Research Topic provide ample implications for future studies that would advance our understanding of the development of eating behaviors and food decision-making. First, the results of studies that examined food choices warrant further investigation on how children develop a reward-oriented or risk-aversive food decision-making pattern that contributes to high energy intake and obesity or food rejection, respectively. Next, studies that examined the role of self-regulation in eating in the absence of hunger suggest that more research is needed to delineate both unique and common effects of various self-regulation skills on the development of healthy and unhealthy eating behaviors. The results of studies that examined parent and peer influences on eating behaviors demand future studies that explore how parent and peer influences increase risks of unhealthy eating and weight gain at a different age, sex, or sociocultural environment. Last, results of intervention studies aimed to improve healthy food choices urge the development of timely and effective prevention and intervention programs bound to scientific research to promote healthy eating and weight management in children and adolescents. Contributions to this Research Topic sheds light on mechanisms underlying the development of eating behaviors and food decision-making in young populations.

## Author Contributions

O-RH wrote the first draft of the editorial. All authors contributed to manuscript review and editing.

## Conflict of Interest

The authors declare that the research was conducted in the absence of any commercial or financial relationships that could be construed as a potential conflict of interest.

## Publisher's Note

All claims expressed in this article are solely those of the authors and do not necessarily represent those of their affiliated organizations, or those of the publisher, the editors and the reviewers. Any product that may be evaluated in this article, or claim that may be made by its manufacturer, is not guaranteed or endorsed by the publisher.

